# Induction of Human Immunodeficiency Virus (HIV-1) Envelope Specific Cell-Mediated Immunity by a Non-Homologous Synthetic Peptide

**DOI:** 10.1371/journal.pone.0001214

**Published:** 2007-11-28

**Authors:** Ammar Achour, Jean-Michel Biquard, Velibor Krsmanovic, Jean-Pierre M'Bika, Damien Ficheux, Marianna Sikorska, Alain J. Cozzone

**Affiliations:** 1 Laboratoire des Interférons, Université Paris Descartes, Centre Universitaire des Saint Pères, Paris, France; 2 Laboratoire de Biologie Moléculaire, Université Claude Bernard, Lyon, France; 3 Peptide Synthesis and Mass Spectrometry, IFR 128 Biosciences Lyon-Gerland, France; 4 Institute for Biological Sciences, National Research Council of Canada, Ottawa, Canada; 5 Institut Biologie et Chimie des Protéines UMR 5086 CNRS/UCBL, Lyon, France; New York University School of Medicine, United States of America

## Abstract

**Background:**

Cell mediated immunity, including efficient CTL response, is required to prevent HIV-1 from cell-to-cell transmission. In previous investigations, we have shown that B1 peptide derived by Fourier transformation of HIV-1 primary structures and sharing no sequence homology with the parent proteins was able to generate antiserum which recognizes envelope and Tat proteins. Here we have investigated cellular immune response towards a novel non-homologous peptide, referred to as cA1 peptide.

**Methodology/Principal Findings:**

The 20 amino acid sequence of cA1 peptide was predicted using the notion of peptide hydropathic properties; the peptide is encoded by the complementary anti-sense DNA strand to the sense strand of previously described non-homologous A1 peptide. In this report we demonstrate that the cA1 peptide can be a target for major histocompatibility complex (MHC) class I-restricted cytotoxic T lymphocytes in HIV-1-infected or envelope-immunized individuals. The cA1 peptide is recognized in association with different MHC class I allotypes and could prime *in vitro* CTLs, derived from gp160-immunized individuals capable to recognize virus variants.

**Conclusions/Significance:**

For the first time a theoretically designed immunogen involved in broad-based cell-immune memory activation is described. Our findings may thus contribute to the advance in vaccine research by describing a novel strategy to develop a synthetic AIDS vaccine.

## Introduction

A major objective of current HIV-1 vaccination strategies is the induction of HIV-1-specific CD8+ MHC class I-restricted CTL responses, suggested to play a pivotal role in viral clearance and protection against HIV-1 disease progression [1]. In effect, the studies from the simian-immunodeficiency virus (SIV) macaque model show that CD8-specific monoclonal antibodies, which block CTL activity, prevent an early viral load reduction [2]–[4]. Also, in monkeys receiving experimental SIV vaccines and subsequently infected with pathogenic SIV, the frequency of mutations within CTL epitopes, presumably responsible for protection failure, correlates with the level of viral replication [5]. More recently, it was reported that a cellular-immunity based vaccine can affect the progression of SIV in rhesus macaques [6]. As shown in earlier experiments with influenza virus, the virus-induced CTLs recognize short peptides derived by proteolytic degradation of viral proteins and selected for presentation at the surface of infected cells by association with MHC class I molecules [7]. However, despite the antigenic complexity of most viruses, the CTL response to viral infection in many instances is dominated by the reactivities directed against a limited number of immunodominant peptidic epitopes. The identities of these epitopes are controlled by the particular Major Histocompatibility Complex (MHC) class I alleles of the host [7], [8]. Furthermore, the marked genetic diversity of HIV-1 and existence of distinct viral subtypes or clades further hinders the development of a universally efficacious HIV-1 vaccine. Thus, genetic variability of HIV-1, especially of the envelope glycoprotein, remains the principal obstacles for elaborating an efficient vaccine against AIDS [9].

Numerous methods, developed on the basis of characteristic periodicities expressed by the discrete Fourier spectra, attempt to introduce the relationship between function and structure of proteins or protein-protein interactions [10]–[24]. They utilize various physico-chemical indices of individual amino acids in a protein sequence, especially those controlling their biological activities, such as biorecognition. For example, a method referred to as the Informational Spectrum Method (ISM, i.e. the EII/ISM concept) [25], [26], also named Resonant Recognition Model (RRM) [27], is based on the estimated indices of electron ion-interaction pseudo potential (EIIP) of delocalized electrons of the amino acid residues [24]. In this method, the sequence of a given protein is converted to a series of integer numbers allowing the identification of spectral periodicities, i.e. characteristic frequencies common for a family of proteins having a similar function [27]–[29]. Such characteristic spectral periodicities have been previously identified for a number of protein families, including interacting proteins such as certain growth factors and their receptor proteins [30]–[33], although the phases corresponding to the common characteristic frequencies of interacting proteins are found to be opposite [27].

Based on the RRM protein-protein recognition concept, we have previously developed the original approach of using non-homologous peptides to induce antibodies against several proteins of different HIV-1 strains, such as gp120 envelope [34] and TAT proteins [35] that are important for HIV-1 replication and modulation of cellular functions. In the previous work [34], we have utilized a pair of synthetic non-homologous 20 amino acid residue peptides, expressing two common characteristic frequencies found by Fourier spectral analysis of CD4 binding fragments of different HIV-1 envelope glycoprotein (gp120) isolates, referred to as A1 peptide (KQQYYWYAWCQPPQDQLIMD) and B1 peptide (DDALYDDKNWDRAPQRCYYQ). We have shown that the B1 peptide, which exhibits the same predicted characteristic frequencies and phases as viral CD4 binding fragments of HIV-1 isolates, is able to induce antibodies cross-reacting with gp120 HIV-1 protein, while the antibodies induced by the A1 peptide, which exhibits the opposite phases to both B1 peptide and gp120 protein at common characteristic frequencies, did not cross-react with gp120 protein [34]. Thus, according to the RRM concept, the pair of A1 and B1 peptides should be considered as interacting peptides because they exhibit the opposite phases at predicted common frequencies derived from the viral CD4 binding fragments of HIV-1 isolates [34].

Following a parallel line of approach, several groups have shown that the open reading frame of the antisense strand DNA may encode “antisense” proteins and that the codons for hydrophilic and hydrophobic amino acids on the sense DNA strand correspond to the complementary codons specifying hydrophobic and hydrophilic amino acids on the antisense strand DNA [36]–[43]. Because of inverted hydropathy, the antisense proteins can sometimes interact with the corresponding sense proteins as demonstrated in the case of mRNA sequences for epidermal growth factor (EGF), transferrin (TF) and interleukin-2 (IL-2), and mRNA sequences for their respective receptors, where a complementarity was found in the region corresponding to the ligand binding portion of the receptors [44]–[46].

In order to generate supplementary peptides as antigenic analogs of both B1 peptide and gp120 envelope proteins of HIV-1, we have combined the EIIP Fourier-based RRM approach with peptide hydropathic properties specified by the complementary DNA strand method. In this manuscript, we describe the immunological properties of a new peptide whose amino acid sequence was deduced from the complementary (antisense) DNA strand to the sense DNA strand encoding the A_1_ peptide, which we called a complementary A1 peptide or cA1 peptide (VHNQLVLRRLAPSVPVVLLF). It should be noted that according to the RRM concept the A1 and B1 peptides could be considered as interacting pair of peptides because they exhibit the opposite phases at predicted common frequencies established for the viral CD4 binding fragments of the HIV-1 isolates [34]. Thus, the “antisense” cA1 peptide was a candidate for an antigenic homolog of both B1 peptide and gp120 envelop proteins of HIV-1. This method was used to induce cellular immunity against HIV-1 and was validated in immunized rabbits as well as in T lymphocytes originated from HIV-1 infected and envelope-immunized individuals. Since rather poor information is available regarding the cross-reactivity of envelope peptides in healthy individuals immunized against the gp160 glycoprotein, we have examined whether the artificially designed cA1 peptide could also be recognized by CTLs derived either from individuals immunized against divergent envelope proteins or from HIV-infected individuals. In addition to the marked cytotoxic response to the cA1 peptide, our results revealed the ability of this artificial peptide to prime *in vitro* the CTLs from gp160 immunized individuals and to generate T cell clones that recognized virus variants. Moreover, the cA1 peptide was recognized by multiple HLA Class I molecules. These findings are discussed with respect to the potential value of the cA1 peptide in the design of an HIV vaccine.

## Materials and Methods

### Generation and synthesis of the non-homologous cA1 peptide

The nucleotide coding sequence of A1 peptide was deduced from the A1 peptide amino acid sequence by selecting the corresponding codons more frequently used in genes highly expressed in *E. coli* [47]. The sequence designated “+” represents sense strand of DNA (or messenger RNA when the base T is replaced by the base U), while the chain designated “-” corresponds to complementary antisense DNA strand, which was used as the coding sequence for the cA1 peptide amino acid sequence, as shown in Table 1.

**Table 1:** Nucleotide coding sequence of A1 and cA1 peptides.

A1 Peptide																					
	K	Q	Q	Y	Y	W	Y	A	W	C	Q	P	P	Q	D	Q	L	I	M	D	
5′[+]	AAA	CAG	CAG	TAC	TAC	TGG	TAC	GCT	TGG	TGC	CAG	CCG	CCG	CAG	GAC	CAG	CTG	ATT	ATG	GAC	3′
3′[−]	TTT	GTC	GTC	ATG	ATG	ACC	ATG	CGA	ACC	ACG	GTC	GGC	GGC	GTC	CTG	GTC	GAC	TAA	TAC	CTG	5′
	F	L	L	V	V	P	V	S	P	A	L	R	R	L	V	L	Q	N	H	V	
cA1 Peptide																					
5′[+]	GTC	CAT	AAT	CAG	CTG	GTC	CTG	CGG	CGG	CTG	GCA	CCA	AGC	GTA	CCA	GTA	GTA	CTG	CTG	TTT	3′
	V	H	N	Q	L	V	L	R	R	L	A	P	S	V	P	V	V	L	L	F	

The sequence designated “+” represents sense strand of DNA while the chain designated “−” corresponds to complementary antisense DNA strand, which was used as the coding sequence for the cA1 peptide amino acid sequence.

Peptide cA1 was synthesized on a Milligen 9050 apparatus with Fmoc-Opfp/Hobt chemistry. The peptide was cleaved in a conventional TFA (trifluoroacetic acid, CF_3_COOH) solution in the presence of scavengers. The final precipitate was solubilized in water and lyophilized. The crude peptide was dissolved in solution A (0.1% TFA in water) and purified on a Vydac column (C18, 5 µm, 25×1 cm) with a gradient over 90 min from 0 to 90% of solution B (70% acetonitrile, 0.09% TFA solution in water) and characterized on an electrospray mass spectrometer (SCIEX API 165) at 2269.4 amu as well as by HPLC (HP 1100) using analytical Vydac column C18 in a gradient over 15 min from 30% to 70% of solution B at a retention time of 14.1 min.

### V3 loop Peptides synthesis and purification

Peptide 18IIIB was synthesized by the solid phase technique at Peninsula Laboratories, Inc. (Belmont, CA) and purified by gel filtration and ion exchange chromatography. The purified peptide yielded a single peak on reverse phase HPLC on C.18 columns with two different solvent systems, trifluoroacetic acid/water/acetonitrile, and 0.05 M NaH_2_PO_4_/acetonitrile and exhibited the expected amino-acid sequence. P18MN was synthesized under the same conditions and was supplied for analytical studies by Dr. J. Berzofsky (National Cancer Institute, Bethesda, MD, USA). P18SIMI peptide was synthesized by Neosystems (Strasbourg, France).

### Monoclonal antibodies

The monoclonal antibodies (mAbs), against human lymphocyte surface markers: CD4 (OKT4), CD3 (OKT3) and CD8 (OKT8), used for phenotypic analysis were purchased from ORTHO Diagnostic Systems (New Jersey, USA).

### HLA typing

HLA transplantation antigens were identified serologically in the Tissue Typing Laboratory at Hôpital Saint Louis (Paris) and Centre National de Transfusion Sanguine (Paris) using a standard complement-mediated cytotoxicity assay.

### Isolation of lymphoid cells

Lymphocytes were isolated from peripheral blood by a Ficoll/Hypaque density gradient method (Seromed, Berlin). All cells were stored in liquid nitrogen after freezing in fetal calf serum containing 10% DMSO.

### Immunization protocols

#### Rabbits

Rabbits were immunized with 300 µg of cA1 peptide combined with complete or incomplete Freund's adjuvant and administered by intradermal injection. The injections (minimum of 8 injections per animal) were administered every 10 days as previously described [29].

#### Humans

Recombinant vaccinia viruses (Lister strain) expressing HIV-1 gp160 (LAV/IIIB, MN and RF strains) were provided by Dr G. Beaud (Institut Jacques Monod, C.N.R.S., Paris, France) and prepared according to the procedure previously described [48], [49]. Soluble recombinant HIV-1 gpl60IIIB was produced in mammalian cells by Drs F. Bex and A. Burny (Université Libre de Bruxelles, Belgium). The recombinant vaccinia virus carrying the env gene of one of SIMI's viruses (clone pULB5384) was used to prime a human volunteer, named SH and to purify the envelope glycoprotein [50].

### Immunized individuals

Donor BI was a healthy HIV-1-seronegative individual who gave an informed consent to participate in this study [51]. Immunizations were performed in agreement with the requirements of the French National Ethics Committee according to the following protocol. He was immunized with recombinant vaccinia virus rV (Lister strain) expressing HIV-1 gp160 (LAV/IIIB strain) given intradermally. He was also boosted with 500 µg of gpl60IIIB as a water-in-oil emulsion injected intramuscularly [51]. Donor SH also gave an informed consent and was vaccinated by a standard intradermal scarification with a stock of recombinant virus expressing SIMI's envelope glycoprotein [52]. Eleven weeks later, donor SH was boosted with intramuscular injection of 150 µg purified gp160SIMI adsorbed on alum as described [52]. The study was approved by the local ethics committee as previously published [51], [52].

### HIV-1-infected individuals

Frozen cells from 17 patients were collected for this study. Informed consent was obtained from all subjects, as reported previously [53]. For T-cell proliferation assays we have used cells from 10 HIV-1-infected donors having CD4+ T cells ranging from 100 to 750/mm3 and plasma virus load varying from 300 to 50.000 HIV-1 RNA copies/ml. These donors were HLA undetermined since they were consulted at Laennec Hospital in Paris. Donors P3-P6 were HIV-1 positive individuals, sharing MHC Class I A2 molecules and exhibiting a strong CTL response against HIV-1 proteins. At the time of the study they began an antiviral treatment and their CD4 number ranged from 350 to 900/mm3.

### Polyclonal generation of CTLs

Human HIV-1-specific lymphocytes were generated in autologous MLC (Mixed Lymphocyte Culture) with PBMC (Peripheral Blood Mononuclear Cells) from donors by mixing 1×10^6^ cells with 2×10^5^ X-ray irradiated (100 Gy) HIV-1-infected autologous phytohemagglutinin-stimulated blasts, where 15–30% of cells expressed HIV-1 antigens [54].

### Peptide induction of CTLs

*In vitro* stimulations of CTLs (4×l0^6^) were performed for 1 hour at 37°C in the presence of 10 µg/ml (1 µg in 100 µl) of cA1 peptide, V3 loop P18IIIB peptide, or V3 loop P18SIMI peptide. After being pulsed by these peptides, the cells were cultured and resuspended at 10^6^ cells/ml. Six days after the initiation, cultures were harvested and the cells (1×10^6^) were re-stimulated with inactivated, but peptide-pulsed autologous PHA blasts cells (4×10^6^ cells, pulsed with 10 µg of peptide) in 2 ml of culture medium supplemented with 20 U/ml recombinant IL-2 (Boehringer Gmbh). Cultures were established in 24 well plates (Falcon, USA) in RPMI1640 medium supplemented with 1 mM sodium pyruvate, 2 mM L-glutamine, 100 u/ml penicillin, 100 µg/ml streptomycin, 10% fetal calf serum (PAA, Austria) and 20 U/ml recombinant IL-2 for 6 to 10 days. Subsequently, the established CTL lines were maintained by weekly stimulation with IL-2-supplemented culture medium [52].

### Inhibition of specific cytotoxicity with monoclonal antibodies

Prior to the initiation of the chromium release assay, effector cells were incubated for 30 min with monoclonal antibodies against CD3, CD4 and CD8 antigens (ORTHO Diagnostic Systems (New Jersey, USA) to a concentration of 10 µg/ml.

### Target cells

Lymphoblastoid cell lines (B-LCL) were generated from the PBMCs of the donors by transformation with the supernatant from B95.8 strain of Epstein-Barr virus (EBV) cultures and were maintained in RPMI1640 medium supplemented with 10% fetal bovine serum.

### Recombinant viruses

Recombinant vaccinia viruses (Lister strain) expressing HIV-1 gp160 (LAV/IIIB, MN and RF strains) were prepared according to the procedure previously described [52]. The recombinant vaccinia virus was used to prime the individual SH and to purify the envelope glycoprotein carried by the env gene of one of SIMI's viruses (clone pULB5384) [50].

### T cell proliferation assay

In animal studies, blood samples were collected from rabbit ears before and after immunization. Lymphocytes were separated on a Ficoll buffy coat and washed twice. The cultures were carried out in 96 well flat bottom plates (Falcon, USA) in RPMI medium supplemented with 1% antibiotics, glutamine, pyruvate and 10% rabbit serum. The rabbit serum collected from the same animal before immunization was heat inactivated for 30 min at 56°C. Cells (2.5×10^5^ per well) were cultured with increasing amounts (1, 10 and 25 µg) of immunogens cA1 peptide, control peptide B1, or HIV-1 gp120 recombinant baculovirus protein. From human subjects, PBMCs were suspended in complete culture medium containing 10% of heat inactivated normal human serum (AB blood type). The cells (2.5×10^5^ per well) treated with medium containing cA1 peptide or control B1 peptide and cultured in an incubator at 37°C containing 5% of CO_2_. Five days after the stimulation, [^3^H] thymidine was added for 18 h, and its incorporation into DNA was measured in a β counter.

### Cytotoxicity assay

EBV transformed B cells (B-lymphoblastoid cell lines, referred to as B-LCL) were infected with recombinant vaccinia virus (rV) or wild-type vaccinia virus at 10 PFU/cell and incubated for 12 hours. These target-cells were then harvested, labelled with 100 µCi (1 Ci = 37 GBq) of sodium [^5l^Cr]Chromate (Amersham) for 60 min at 37°C, washed three times before being resuspended to the appropriate concentration (5×10^3^ cells per well) for the use as target cells [52]. For peptide testing, the cells were pulsed with 10 µg/ml of peptide [52] before labeling and lysis by the effector cells was measured in a 4 hour chromium-release assay performed in triplicate. The percentage of specific cytotoxicity was determined using the formula: 100×[(release in assay-spontaneous release)/(maximum release-spontaneous release)]. The maximum release was determined by lysis of target-cells in 1 N HCl. Spontaneous chromium release in medium alone ranged from 10 to 20%.

## Results

### T-cell proliferation response

#### Immunized rabbits

**Figure 1:**
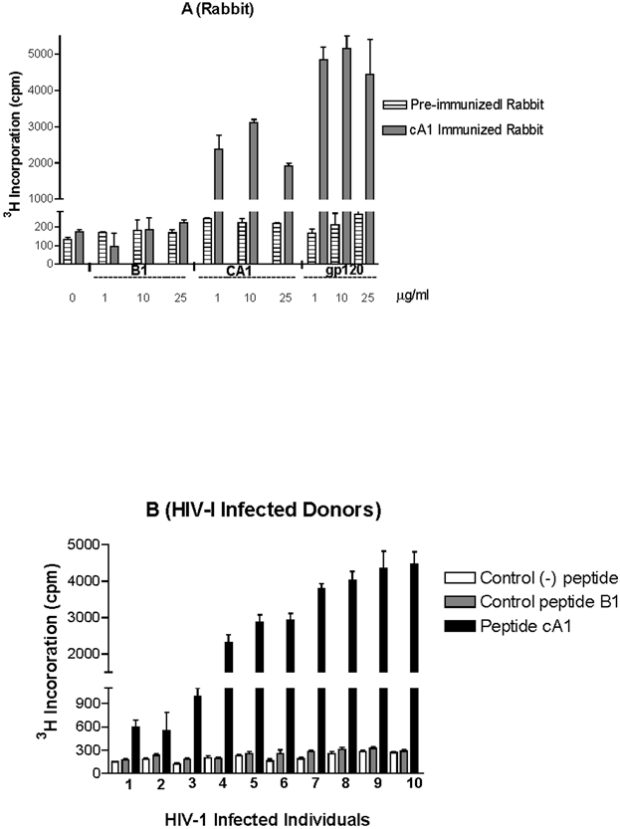
The cA1 peptide-induced T cell proliferation. (A) Proliferation response in cA1 peptide-immunized rabbits. Lymphocytes collected before and after immunization were stimulated with various concentrations of control B1 peptide, cA1 peptide or recombinant gp120 protein added to the culture media. (B) Proliferation response of PBMCs from HIV-1-infected individuals. Cells were obtained from 10 HIV-1-infected individuals (1-10) and their proliferation response was measured after stimulation with 1 µg/ml of control B1 peptide, cA1 peptide or after incubation without (-) peptide, as indicated. The incorparation of [^3^H]thymidine into DNA was measured 6 days after the stimulation. Tests were performed on triplicate samples.

In cA1-immunized animals we have observed a strong stimulation of lymphocytes sensitized by either peptide cA1 or HIV1 gp120 recombinant protein, reaching a highest proliferative activity at 1 µg/ml, as compared to cells cultured with control B1 peptide (Fig 1A). The stimulation of lymphocyte proliferative activity was higher in the presence of gp120 protein when compared to the effect of cA1 peptide. By contrast, no significant proliferation occured in lymphocytes before immunization. These results indicated that immunization of rabbits with the artificial cA1 peptide could induce a cellular response similar to HIV-1 gp120 protein. The possibility that the cA1 peptide might be recognized by human immune cells in HIV-1 infected individuals was further tested.

#### HIV-1-infected individuals

We have then stimulated the PBMCs, obtained from 10 randomly selected naturally infected patients, with the cA1 peptide for 6 days and evaluated the ability of T cells to proliferate using [^3^H] thymidine incorporation into DNA. As shown in Fig. 1B, the lymphocytes were able to proliferate in the presence of cA1 peptide, while no significant specific proliferation was observed when cells were incubated with the control B1 peptide. It is noteworthy that T cells derived from healthy HIV-1-seronegative individuals did not initiate proliferation in the presence of cA1 peptide (data not shown), suggesting that the cA1 peptide recognition might have been processed after the viral infection. No more cells were available to precise the nature of the proliferating cell population. Although at the time of the study, no HLA typing (Class I and II) was provided from the infected donors, the lymphocyte proliferative activity of some cells in response to cA1 peptide may have had a CTL response.

### Cytotoxic T-cell response in HIV-1-infected individuals

**Figure 2:**
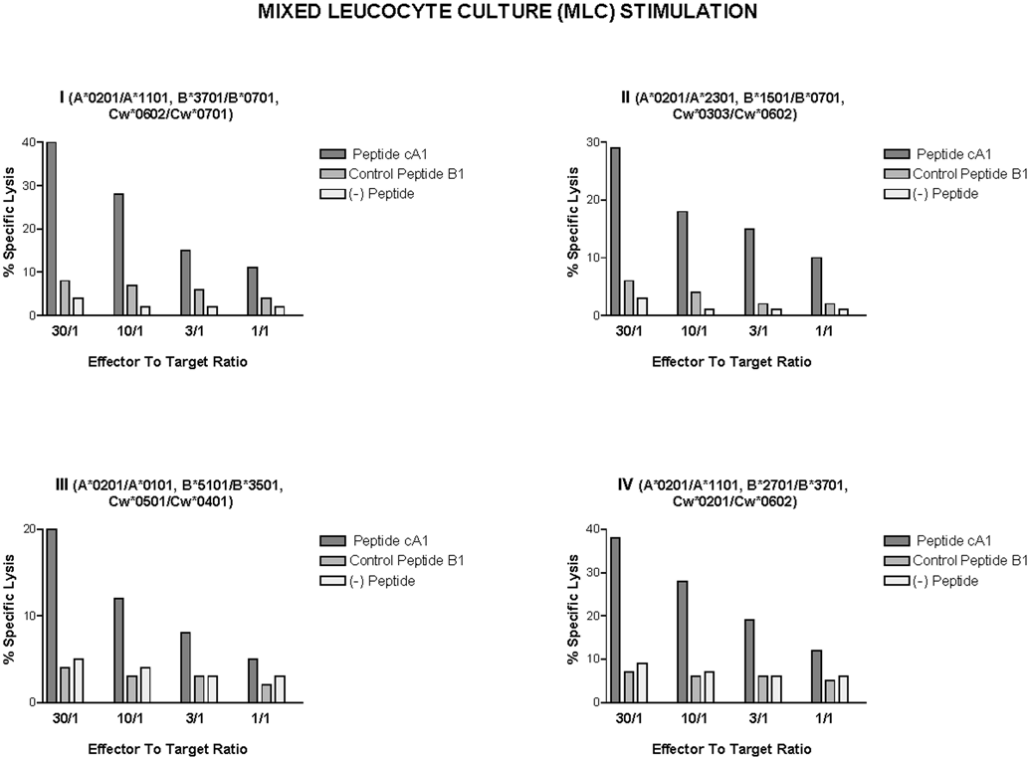
CTL activities derived from cell cultures of 4 HIV-infected individuals (I, II, III and IV) directed against autologous target-cells presenting cA1 peptide, control B1 peptide, or target-cells infected with either wild type vaccinia virus or recombinant vaccinia virus expressing gp160MN. The CTL cultures were generated by polyclonal activation with the irradiated autologous cells. After a 10 day incubation the effector cells were tested for CTL activity as described in materials and methods. The data are representative of 3 independent experiments performed in triplicates. Standard errors of the mean (s.e.m) from triplicate wells were 5% of the mean.

Evaluation of cytotoxic T cells in HIV1-infected individuals is generally achieved after a necessary step of *in vitro* stimulation. As reported previously, HIV-specific CTLs were generated in mixed leucocyte culture (MLC) with irradiated HIV-1-infected autologous stimulated blasts and used as effector cells in the assay [49]. Autologous lymphoblastoid cells were used as target-cells, which were infected either with wild-type vaccinia virus or recombinant gp160MN vaccinia virus, or pulsed with cA1 peptide or control B1 peptide. The results of each subject (1–4), identified by HLA Class I status, are presented in Figure 2. No specfic lysis was obtained when target-cells were infected with wild vaccinia virus or coated with B1 peptide. By contrast, a cytotoxic response towards target-cells expressing gp160MN protein (the latter being representative of the most prevalent strains in Europe and North America) or pulsed with the cA1 peptide, was observed. These results indicate that the cA1 peptide could be recognized by HIV-1-specific CTLs generated following standard activation.

**Figure 3:**
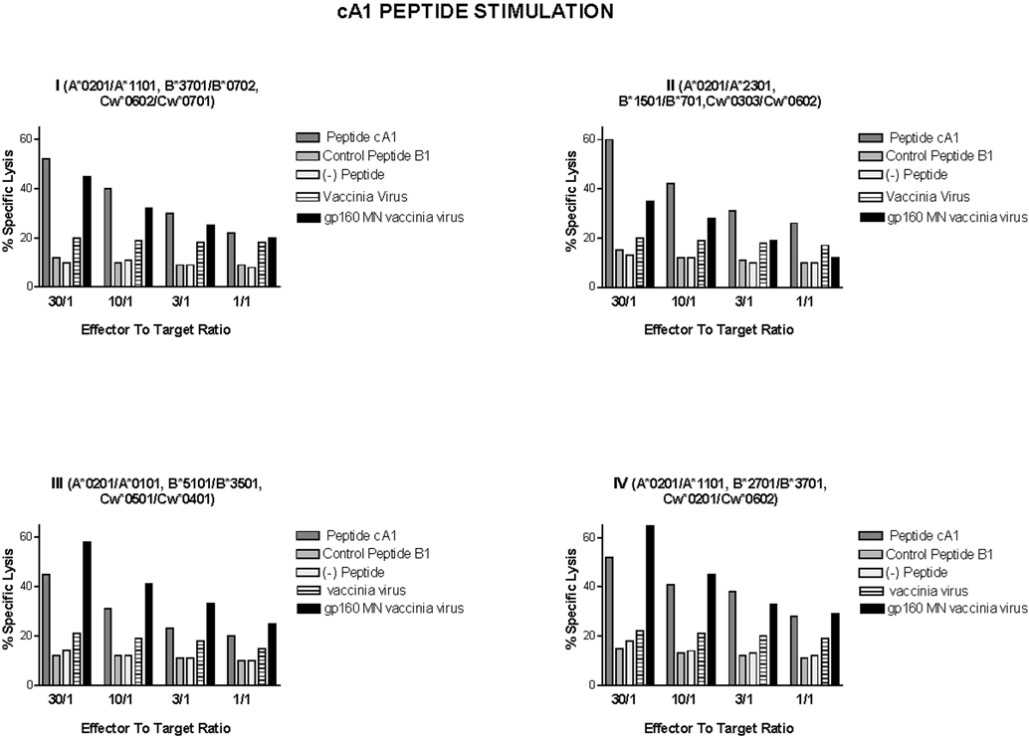
CTL activity of cA1-stimulated cell cultures of 4 HIV-infected individuals (I, II, III and IV) directed against autologous target-cells presenting cA1 peptide, control B1 peptide, or target-cells infected with either wild type vaccinia virus or recombinant vaccinia virus expressing gp160MN. The secondary cultures of the effector cells were obtained by incubating the PBMCs with the cA1 peptide and their CTL activity was tested after 10 days of incubation. The data are representative of three independent experiments performed in triplicates. Standard errors of the mean from triplicate wells were < 5% of the mean.

Since the cA1 peptide was processed for recognition by HIV-1-specific CTLs, we have evaluated the capacity of this peptide to stimulate env-specific T cell memory from AIDS patients. Thus, CTLs were generated in MLC with irradiated cA1-pulsed autologous stimulated blasts. The results, shown in Figure 3 illustrate that autologous target-cells expressing env antigen or presenting cA1 peptide were killed very effectively by cA1-peptide stimulated effector cells. This specific elimination of target-cells appeared significantly higher when compared to the standard activation assay (see Figure 2). By contrast, no specific killing was observed by lymphocytes stimulated, under the same conditions, by the control B1 peptide (data not shown).

It should be noted that all 4 individuals studied shared the HLA Class I A*0201 molecule suggesting that the cA1 peptide might share some common features with the MHC class I binding peptides. To establish that we have explored the **SYFPEITHI** database of T-cell epitopes and MHC ligands [55], typically used to facilitate the search for binding peptides and the prediction of T-cell epitopes [56]. As shown in Table 2, the cA1 peptide had both preferred and auxiliary residues to be presented by the HLA-A*0201 molecule, exhibiting a score at 29, compared to the well-known epitope GILGFVFTL derived from the influenza protein, which scores 30. By comparison, the control B1 peptide had only auxiliary residues to be presented by the A*0201 molecule and scores a maximum of 12. More interestingly the cA1 peptide presents overlapping sequences (5–14; 9–18) to be presented to HLA-A*0201 molecule as either nanomers or decamers.

**Table 2:** Sequence motifs important for peptide binding to the human MHC class I molecule HLA-A*0201and cA1 peptide structure using SYFPEITHI database.^a^

V	H	N	Q	L	V	L	R	R	L	A	P	S
1	2	3	4	5	6	7	8	9	10	11	12	13
V	P	V	V	L	L	F						
14	15	16	17	18	19	20						

HLA-A*0201 nanomers			HLA-A*0201 decamers		
Position	Sequence	Score b	Position	Sequence	Score b
9	R **L** A P S V P V **V**	29	9	R **L** A P S V P V **V** L	26
6	V **L** R R L A P S **V**	25	10	**L** A P S V P V **V** L L	22
11	A **P** S V P V V L **L**	19	5	L **V** L R L L A P **S** V	21
10	L **A** P S V P V V **L**	18	8	R **R** L A P S V P **V** V	17
8	R **R** L A P S V P **V**	15	1	V **H** N Q L V L R **R** L	16
2	H **N** Q L V L R R **L**	14	4	Q **L** V L R R L A **P** S	16
5	L **V** L R R L A P **S**	14	7	L **R** R L A P S V **P** V	14
4	Q **L** V L R R L A **P**	12	6	V **L** R R L A P S **V** P	11
1	V **H** N Q L V L R **R**	8	11	A **P** S V P V V L **L** F	7
3	N **Q** L V L R R L **A**	7	2	H **N** Q L V L R R **L** A	4
12	P **S** V P V V L L **F**	4	3	N **Q** L V L R R L **A** P	2
7	L **R** R L A P S V **P**	2			

a HLA-A2-binding peptides are 8–10 amino acid (aa) residues long, with a Leu at position 2 (P2), and a Val or Leu at position 9. However, other peptide side chains (bold) contribute to the stability of the interaction. In certain cases, the optimal length for peptide binding can be longer than 9 residues.

b The allocation of values is based on the frequency of the respective amino acid in natural ligands, T-cell epitopes, or binding peptides.

### Analysis of the CTL response in gp160 vaccinated recipients

**Figure 4:**
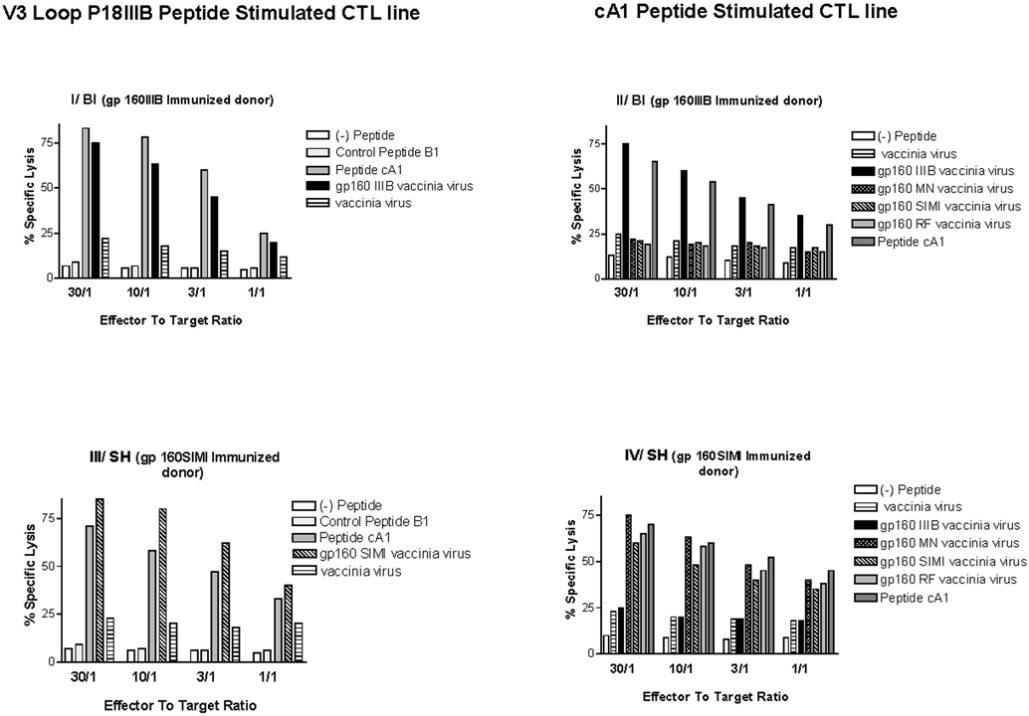
CTL activity of effector cells derived from the BI donor vaccinated with IIIB-env-recombinant protein and from the SH donor vaccinated with SIMI-env-recombinant directed against autologous target-cells presenting cA1 peptide and HIV-1 env proteins. The secondary cultures of the effector cells were generated *in vitro* after incubation with V3 loop P18 peptide ( panels I, III) and cA1 peptide (panels II, IV). The data are representative of four independent experiments performed in triplicates. Standard errors of the mean from triplicate cultures were all <7% of the means.

To evaluate the CTL response induced by the recombinant vaccinia-env/gp160 regimen in human subjects, we performed *in vitro* stimulation with V3 loop P18 peptide to activate and expand the env-specific memory T cells in peripheral blood from the vaccine recipients, as previously described [52]. Based on the sequence analysis (Table 2), the cA1 peptide contained the anchor residues for association with the HLA class I A*0201 molecule, therefore, it could be recognized by the env-specific memory T cells. Thus, cells were obtained from a healthy individual BI, who was immunized with the recombinant vaccinia virus expressing the HIV-1IIIB envelope gene and boosted with the purified recombinant gp160IIIB protein (a strain, which did not cross-react with divergent RF or America-Europe consensus MN strains) as well as the cells from the donor SH, who was vaccinated with recombinant vaccinia virus expressing SIMI envelope glycoprotein cross-reacting with America-Europe consensus strain and was boosted with SIMI gp160 protein. The cytolytic responses to the HIV-1 envelope were tested 4 weeks after the last boost (51). The immune cells from the donor BI were further stimulated *in vitro* with P18IIIB in the presence of IL-2. These CTLs were able to kill the autologous B-EBV LCL infected with recombinant vaccinia virus expressing the whole gp160IIIB, but not the control target-cells infected with wild-type vaccinia virus (Fig. 4, panel I). They also killed the autologous B-EBV LCL pulsed with cA1 peptide, while the cells pulsed with control peptide B1 survived (Fig. 4/I). The CTLs derived from the donor SH and stimulated *in vitro* with P18IIISIMI peptide were able to lyse the autologous B-EBV LCL infected with recombinant vaccinia virus expressing the whole gp160SIMI protein, but not the control target-cells infected with wild-type vaccinia virus. Moreover, targets pulsed with the cA1 peptide were also killed (Fig. 4 panel, III).

Subsequently, we have evaluated the capacity of cA1 peptide to stimulate envelope-specific CTLs in human subjects immunized with recombinant vaccinia-env/gp160 proteins. The cA1 peptide was used to activate and expand env-specific memory T cells in lymphocytes from the vaccine recipients. Following 10 days of stimulation *in vitro*, the responding T cells were assayed for specific cytotoxicity. The CTLs from the donor BI, stimulated with cA1 peptide in the presence of IL-2, were able to kill the autologous B-EBV LCL infected with recombinant vaccinia virus expressing the whole gp160IIIB as well as the autologous B-EBV LCL pulsed with cA1 peptide (Fig. 4 panel II). They did not kill, however, the vaccinia virus infected target-cells expressing either gp160MN, gp160SIMI, or cells infected with wild vaccinia virus (Fig. 4/II).

The CTLs from the donor SH, generated following activation with the cA1 peptide, also developed the capability to kill target-cells pulsed with the cA1 peptide (Fig. 4, Panel IV)). Most interestingly, these effector cells were able to kill target-cells infected with vaccinia virus expressing gp160 protein of the MN strain, which may be regarded as representative of HIV-1 strains prevalent in North America and Europe, as well as SIMI and the highly divergent RF isolate strains. On the other hand, the target-cells infected with recombinant vaccinia virus expressing IIIB/LAI gp160 as well as target-cells infected with wild-type vaccinia virus were insensitive to these CTLs from the donor SH (Fig. 4, panel IV).

### Determination of the class I MHC restriction of the cA1 peptide CTL determinant in gp160-immunized individuals

**Figure 5:**
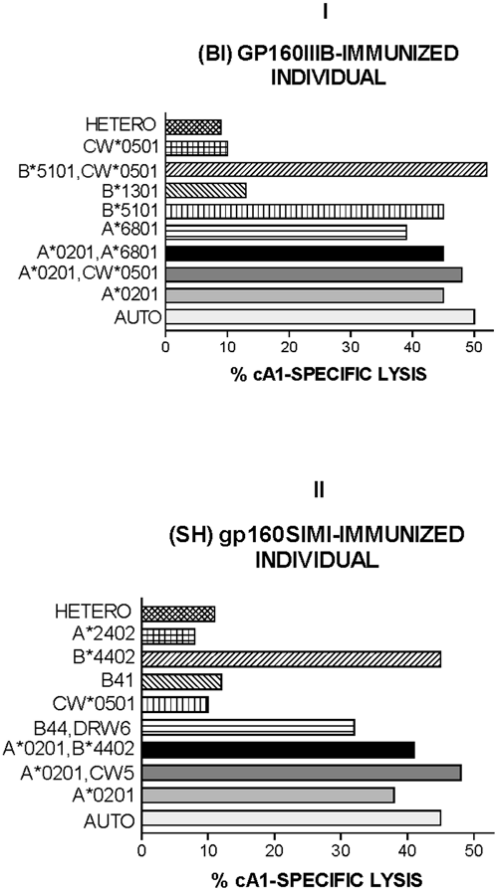
The cA1 peptide-specific cytotoxicity of lymphocytes originating from gp160IIIB-immunized individual BI (HLA-A*0201, -A*6801, -B*5101, -B*1301, -CW*0501) (panel I) and from gp160SIMI-immunized subject SH (HLA-A*0201, -A*2402, -B*4402, -B41, -CW*0501, -DRB1*13, -DR-) (panel II ), both being HLA- A*0201 Class I restricted. Autologous EBV-LCL (AUTO), heterologous (HETERO) or cells sharing the HLA molecule with the donor were sensitized by cA1 peptide. Percentage of specific lysis was determined at the effector to target (E/T) ratio of 10:1. The assays were performed in triplicate with 5000 target-cells/well.

The CTL cell lines were generated using a second stimulation with irradiated, autologous PHA blasts pulsed with the reactive cA1 peptide. The effector cells from gp160IIIB immunized donor BI (HLA-A*0201, -A*6801, -B*5101, -B*1301, -CW*0501) were assayed for cytotoxicity on EBV-transformed cells. To determine the MHC restriction, we have analyzed the cA1 peptide-specific lysis towards target-cells, such as autologous B-LCLs, HLA class I heterologous B-LCLs or B-LCLs, expressing the HLA-A*0201 molecule (Fig. 5/I). The data revealed that the recognition of cA1 peptide by effector cells specifically occurred in association with the HLA-A*0201 molecule. Most interestingly, we have found that the cA1 peptide was recognized by CTLs in association with HLA-A*6801 and -B*5101 molecules, but not in association with HLA-B*1301 and HLA-CW*0501.

Subsequently, we determined the class I MHC restriction of the cA1 peptide CTL determinant in cells derived from SIMI gp160-immunized individual SH (Fig. 5/II). Using a panel of B-EBV transformed cells, we found that these specific CTLs recognized the cA1 peptide by the A*0201 class I MHC molecule, the same restriction element as the CTLs derived from gp160IIIB immunized donor BI. Thus, these CTL lines specifically lysed the autologous (HLA-A*0201, -A*2402, -B*4402, -B41, -CW*0501, -DRB1*13, -DR-) lymphoblastoid cells pulsed with cA1 peptide, while heterologous B-LCLs (HLA-A*01, A-, -B*0801, -B*1401, -CW*0701, -C-, -DR-, -DRW-) cells, also pulsed with cA1 peptide, were not lysed. The lysis was observed in target-cells in which HLA-A*0201 and HLA-B*4402 were the only shared restricting elements. Furthermore, the cA1 peptide was not recognized by the effector cells in association with HLA-A*2402, HLA-B41, HLA-CW*0501 and HLA-DRB1*13. Therefore, these results indicated that the class I HLA-A*0201 and -B*4402 molecules were restrictive elements for the cA1 peptide in immunized individual SH.

### Determination of the phenotype of cA1-specific effector cells

**Figure 6:**
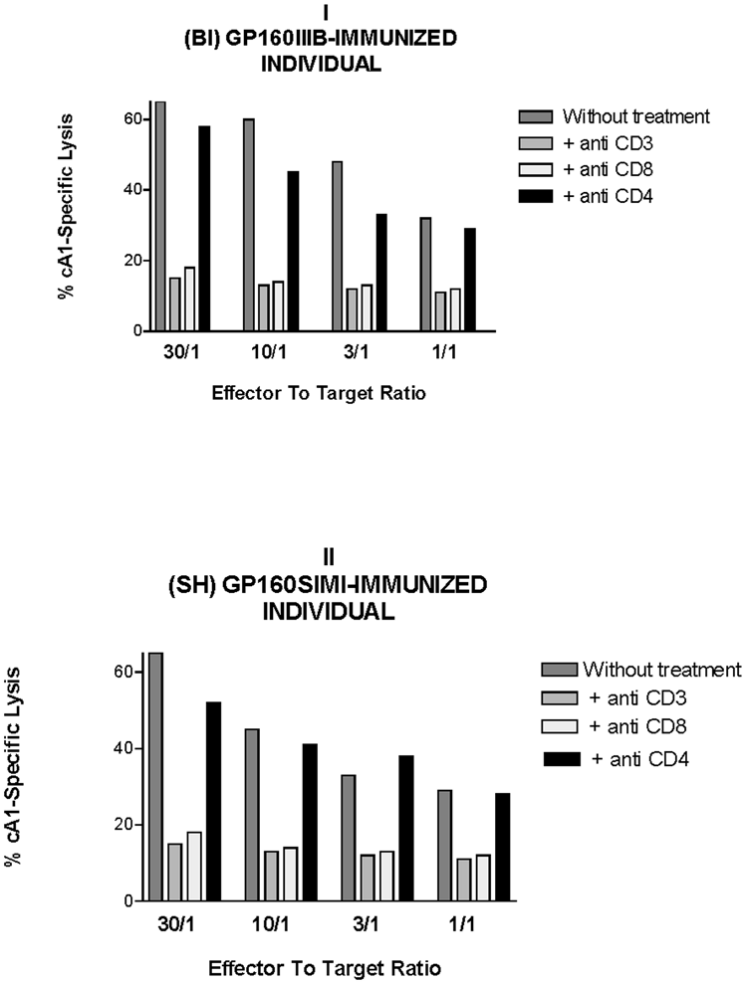
Phenotype of cA1 peptide-specific CTLs derived from gp160IIIB-immunized individual BI (panel I) and from gp160SIMI-immunized subject SH (panel II). Autologous B-LCLs, sensitized by cA1 peptide, were cultured with effector cells pre-treated with anti-CD8, anti-CD4 or anti-CD3 monoclonal antibodies. The control group was not treated with antibodies. Standard errors of the mean, from triplicate wells, were less than 5% of the mean.

Since immunization might have primed CD4+ CTLs, we determined the phenotype of the specific CTLs stimulated with the cA1 peptide and derived from gp160IIIB (donor BI) and gp160SIMI (donor SH) immunized individuals (Fig. 6). Accordingly, we established that the cA1 peptide-specific CTLs were conventional CD4- CD8+ CD3+ lymphocytes, because the specific lysis was abrogated by the treatment with monoclonal antibodies to CD8 and CD3 surface markers, but not with antibody to CD4 molecule (Fig. 6).

## Discussion

T cells that mediate cellular immunity can detect the presence of intracellular pathogens, because infected cells display on their surface peptide fragments derived from the pathogen's proteins, so that T cells recognise the peptide–MHC molecule complex and kill the infected cells. Thus, the MHC class I molecules deliver peptides, originating in the cytosol, to the cell surface that are recognised by CD8 T cells, while the MHC class II molecules deliver peptides originating in the vesicular system to the cell surface, where they are recognised by CD4 T cells. Previously, we have reported that a non homologous synthetic peptide, selected with techniques based on pattern property algorithm procedures, can induce humoral response directed against the HIV-1 gp120 as well as against the Tat proteins [34], [35]. In the present study, we have described a novel peptide, referred to as peptide cA1, designed using the inverted hydropathy, i.e. complementary [antisense] strand DNA method (as described in Introduction and Material and Methods), which generated a cellular response towards the recombinant gp120 protein in cA1 peptide-immunized rabbit. Moreover, *in vitro* this peptide generated both the T cell proliferation and CTL response in cells derived from either naturally HIV-1 infected individuals or envelope-immunized donors. Significantly, our data showed that the MHC-restricted, peptide-specific CTLs were easily generated *in vitro* by the artificial cA1 peptide alone, and that these CTLs were capable of lysing target-cells expressing endogenous antigens in naturally infected patients and in healthy gp160-immunized individuals. Therefore, it is possible that the high frequency of env-specific CTL in cultures not only reflects the high level of memory specific CTL among PBMCs, but also a rapid expansion of these gp160-specific killers in response to stimulation with cells presenting the cA1 peptide.

The cA1 peptide was also able to induce T helper cell response in both cA1-immunized rabbits and HIV-infected individuals, as demonstrated by cell proliferation assays. However, it remains unclear whether this response reflected a threshold for intracellular signalling or more complex events such as the action of co-stimulatory molecules on the antigen presenting cells. The T helper cells are also involved in the induction of CTLs by responding to epitopes presented by the MHC class II molecules. Although the T helper cells and CTLs generally respond to different epitopes, the cA1 peptide could also induce T helper cells. Therefore, it may be postulated that the dual recognition of the cA1 peptide by both class I and II molecules contributed to its efficient recognition. Despite the antigenic complexity of most viruses, the CTL response to viral infection is, in many instances, dominated by the reactivity directed against a limited number of epitopes and their identity are rather controlled by the particular MHC class I alleles of the host.

Although the HLA selectivity remains a problem for the eventual vaccination with peptide sequences, we have found that the cA1- peptide specific CTLs were restricted by more than one class I molecule, including HLA-A*0201, -A*6801, -B*4402 and HLA-B*5101 molecules. Our results are in accordance with the **SYFPEITHI** database indicating effectively that cA1 peptide shares anchor residues to be presented by HLA- A*0201, -A*6801, -B*4402 and HLA-B*5101 molecules, the HLA-A*0201 family being the largest and most diverse allele family at the HLA-A locus. It is established that nine amino acids of the antigenic peptide interact strongly with the MHC binding site, while two A2-supermotifs are already defined in the literature [57]. As indicated in the previous studies, most HLA-A*0201-binding peptides are 9 amino acid residues long, with preferred aliphatic residues at position 2 (P2) for Leu and for Val or Leu at position 9 (P9). Although not all nine amino acids interact strongly with the MHC binding site, all of them contact and make significant interactions with the MHC molecules [57]. Six of the peptide amino acids fall into the pockets of the binding site; they are defined as primary (positions 2 and 9) and secondary (positions 1, 3, 6 and 7) anchor positions. The remaining three amino acids are accessible to solvent and can also interact with receptors on the T cell, being able to affect MHC binding affinity in several ways - through direct non-bonded interactions with the MHC, by causing conformational changes in anchor residues and by altering the dynamic properties of the whole peptide. However, further studies are necessary to demonstrate whether or not the cA1 peptide can be recognized in association with additional HLA class I and class II allotypes.

Our study also points out a major problem in HIV-1 vaccine development, which is antigenic sequence heterogeneity. The ability of a vaccine to induce the cross-reactive CTLs, with a wide range of HIV-1 isolates, is of a great importance. Although the hypervariability of the gp160 protein raises concern for the design of a vaccine, it is encouraging that the cA1 peptide was able to prime the CTLs against both RF and MN virus variants, the latter being a representative of the most prevalent strains in Europe and North America. Whatever is the mechanism involved, a broad-based vaccination protocol producing group-specific CTLs is thus a mandatory prerequisite for understanding a vaccination program. Our observations suggest that the design of a vaccine should take into account the selection of a broad array of epitopes from different strains to confer a broad-based immunity (52).

Finally, this study further substantiates the concept that rational design of various T-cell epitopes may lead to stronger peptide immunogens. This strategy would allow the selection of beneficial peptides recognizing a variety of viral proteins and/or strains in association with multiple MHC class I alleles, excluding viral epitopes responsible for the deleterious immune responses. The findings reported here may be useful for developing an effective vaccine designed to induce the cell-mediated immunity against HIV-1.
